# Association Between Over-the-Counter Magnesium Supplement Use and Health Outcomes in Veterans with Newly Diagnosed Heart Failure

**DOI:** 10.3390/nu17233687

**Published:** 2025-11-25

**Authors:** Yan Cheng, Andrew R. Zullo, Ying Yin, Yijun Shao, Senait Tekle, Simin Liu, Qing Zeng-Treitler, Wen-Chih Wu

**Affiliations:** 1 Department of Clinical Research and Leadership, George Washington University, Washington, DC 20037, USA; yan_cheng@gwu.edu (Y.C.); yiny@email.gwu.edu (Y.Y.); yshao@email.gwu.edu (Y.S.); stekle@email.gwu.edu (S.T.); zengq@email.gwu.edu (Q.Z.-T.); 2 Washington DC VA Medical Center, Washington, DC 20422, USA; 3 Transformative Health Systems Research to Improve Veteran Equity and Independence (THRIVE) Center of Innovation, VA Providence Healthcare System, Providence, RI 02908, USA; andrew_zullo@brown.edu; 4 Department of Epidemiology, Brown University, Providence, RI 02903, USA; 5 Department of Epidemiology & Biostatistics, University of California, Irvine, CA 92617, USA; simin.liu@uci.edu; 6 Department of Medicine, Brown University, Providence, RI 02912, USA

**Keywords:** heart failure, magnesium, dietary supplements, target trial

## Abstract

Background: Individuals with heart failure (HF) are at increased risk of magnesium deficiency. Magnesium supplements are widely available and being used without clear evidence of efficacy in HF. Methods: We emulated a target trial to assess the association between magnesium supplements and adverse outcomes in U.S. veterans with newly diagnosed HF. Eligible patients were outpatients who received ambulatory care in the Veterans Health Administration between 1 January 2000 and 31 December 2020. Veterans with a hospitalization within 30 days prior to the eligible date, previous magnesium supplement or replacement use, or end-stage renal disease were ineligible for the trial. Initial self-reported magnesium supplement use (measured at eligible date) was identified in medical records using natural language processing and then checked repeatedly to confirm continuous use. The outcome was all-cause hospitalization or death. Patients were followed for up to five years from the eligible date and were censored if they changed from the assigned treatment strategy or initiated prescribed magnesium replacement. We applied inverse probability treatment weighting and Cox’s regression to estimate hazard ratios (HRs), with sensitivity analyses in patients surviving ≥ 6 months and those with continuous documentation of magnesium supplement use. Results: We enrolled 9900 magnesium supplement users and 9900 matched non-users. In the weighted cohort (mean age 72.6 years; 12.6% African American; 3.4% women; median follow-up 0.7 years), users had significantly better survival in both primary and sensitivity analyses (HR in primary analysis: 0.81 [0.77–0.86], *p* < 0.0001; HRs in sensitivity analyses: 0.91 [0.85–0.97], *p* = 0.0025 and 0.77 [0.72–0.82], *p* < 0.0001, respectively). Conclusions: magnesium supplement use was associated with a reduced risk of all-cause mortality or hospitalization among veterans with HF.

## 1. Introduction

Magnesium is an essential intracellular mineral involved in numerous key enzymatic processes in the human body [1] and plays a critical role in preventing arrhythmia, improving hemodynamics, and supporting cardiovascular function [2,3]. However, individuals with heart failure (HF) are at increased risk of magnesium deficiency [4]. Findings from two studies suggest that low magnesium dietary intake and low plasma magnesium levels are linked to increased risk of HF-related hospitalizations [5,6]. Additionally, a low plasma magnesium level was also associated with a higher risk of cardiovascular death (hazard ratio [HR]: 1.38; 95% confidence interval [CI]: 1.04–1.83; *p* = 0.024) in 1569 patients with chronic HF [7]. However, the literature does not suggest that dietary intake alone relates to changes in cardiovascular outcomes. Our previous study showed that dietary magnesium intake was not related to mortality in women with HF [8]. Another study showed that although low serum magnesium was associated with a higher risk of arrhythmias, there was no observed association between dietary magnesium intake and arrhythmia risk [9]. Interestingly, magnesium supplementation has been shown to reduce the risk of cardiac arrhythmias in patients with HF in two randomized controlled trials (RCTs) [10]. But it remains unknown if magnesium supplementation has an effect on all-cause mortality or hospitalization in patients with HF.

To assess the effects of magnesium supplementation, rigorously designed RCTs are ideal. However, RCTs are often expensive for a nutritional supplement, as they typically require large participant cohorts and extended follow-up periods to detect relatively small effect sizes [11,12,13]. Target trial emulation is designed to replicate the structure of an RCT on the observational data to evaluate clinical effectiveness while controlling the biases inherent to observational research [14,15,16,17]. This study aimed to assess the association between magnesium supplementation and all-cause hospitalization or death using a target trial emulation.

## 2. Material & Methods

### 2.1. Study Population

Study subjects were patients newly diagnosed with HF between 1 January 2000 and 31 December 2020, identified from the US Veterans Health Administration (VHA) national electronic health records (EHR). HF was defined based on the International Classification of Diseases (ICD) codes (ICD9: 398.91, 402.01, 402.11, 402.91, 404.01, 404.03, 404.13, 404.91, 428; ICD10: I11.0, I13.0, I13.2, I50). The diagnoses were identified from both inpatient and outpatient encounter data in the VHA system.

### 2.2. Study Design and Ethics

This was a retrospective cohort study. The study was approved by the VHA Central Institutional Review Board (IRBNET#1710776-1, 23 April 2021), which granted a waiver of informed consent. Due to the sensitive nature of the data involved, access to the dataset is restricted; qualified researchers trained in human subject confidentiality may request access by contacting the Washington DC VA Medical Center via email at helen.sheriff@va.gov.

### 2.3. Target Trial Emulation

We emulated a target trial by “enrolling” eligible patients into the study cohort. The elements of the target trial and the emulation are summarized in Table 1. A patient would be qualified for inclusion in a trial if, on an outpatient clinical note date, they met the following criteria: (1) had the initial HF diagnosis within 1 year before, (2) had at least two outpatient clinical encounter notes within the previous 12 months (to confirm magnesium supplement use status), (3) had no records of prescribed or supplemental magnesium use prior to this date documented in the electronic medical records (EMR), (4) had no hospitalization within 30 days before or at the eligible date, and (5) had no end-stage-renal-disease (ESRD) any time before or at the eligible date. The purpose of requiring “no hospitalization within 30 days before or on the eligible date” was to exclude patients who were acutely ill, since these patients would be more likely to die earlier and have less opportunity to initiate magnesium supplements, thereby further reducing selection bias.

### 2.4. Exposure

The primary exposure of interest was EMR documentation of the patients’ current use of over-the-counter magnesium supplements, as documented in outpatient clinic visit notes. We identified this information using natural language processing (NLP) techniques [18] developed by our team to extract mentions of magnesium supplement use from the clinical notes. The NLP process and performance in identifying magnesium supplement use is described in detail elsewhere [18]. Validation of the NLP method used for dietary supplement and vitamin use showed an AUC of 98.9%, a precision of 96.2%, and a recall of 87.7% [18].

Eligible date: The status of magnesium supplement use on the date of the outpatient clinic visit when a patient became eligible for the target trial is the eligible date (index date, time 0). That is also the date the patient was classified as a magnesium supplement user or non-user (Table 1).

Exposure status during follow-up: For both groups, any patient without clinic visit notes for more than one year was considered lost to follow-up and was censored one year after their last documented clinic visit. For magnesium supplement users, if magnesium supplement use was not mentioned in the notes for over one year, the magnesium supplement use was considered discontinued one year after the last recorded use. If a patient was initially classified as a non-user of magnesium supplement but a clinic note during follow-up indicated magnesium supplement use, this was treated as a change in exposure status, and the patient would be censored at that time point.

To maximize the sample size of magnesium supplement users, we included all patients who were qualified to be a user. Then, we matched a randomly selected group of controls who had a clinic visit that was on the same date as a user in a 1:1 ratio.

**Figure 1:**
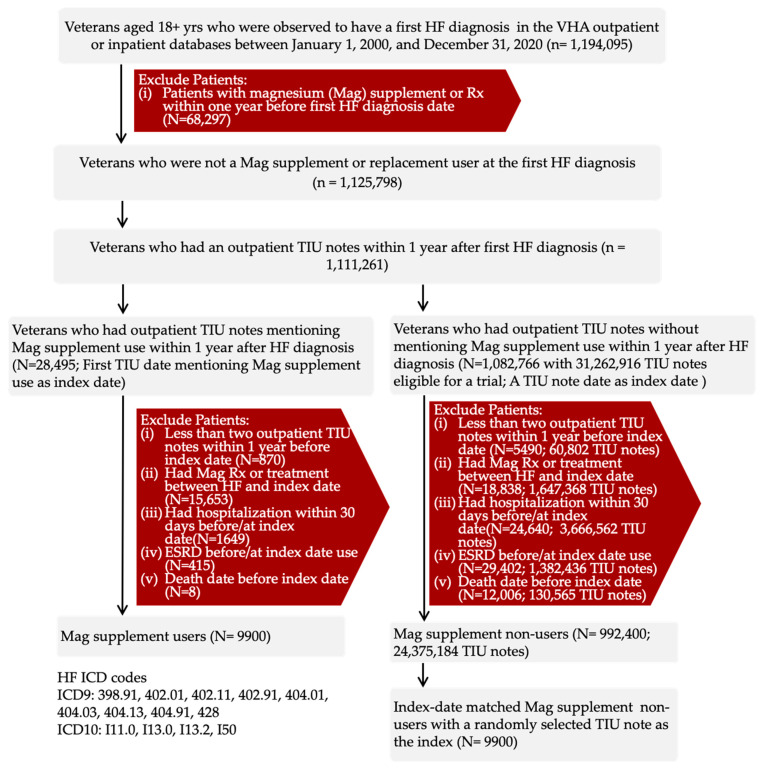
Cohort Assembly.

**Figure 2:**
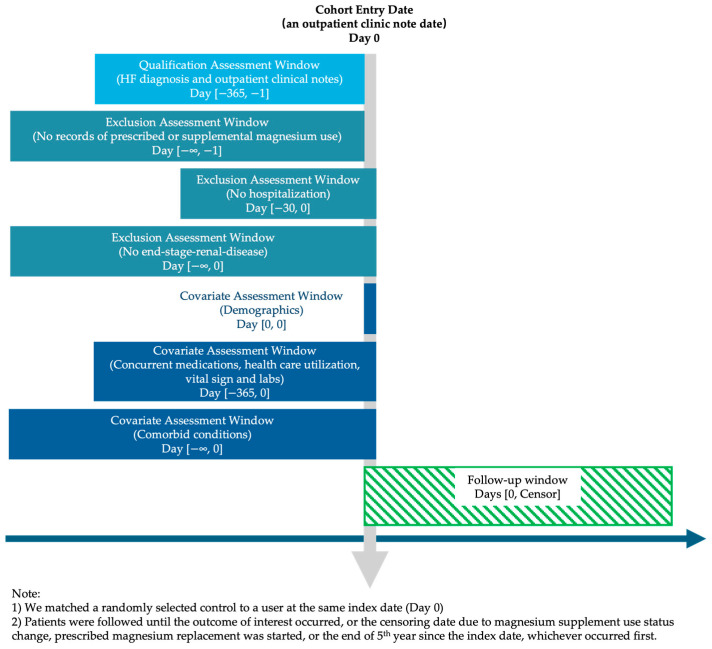
Graphical depiction of study design.

The process of the target trial emulation is displayed in Figure 1. The graphical depiction of the study design is displayed in Figure 2. As described earlier, both groups were required to meet the same eligibility criteria. We used the same approach to identify exposure status and define the index date. Regardless of whether participants were users or non-users, the follow-up began on the eligibility date. All these measures were implemented to control for immortal time bias.

### 2.5. Study Outcomes and Follow-Up

The main study outcome was all-cause hospitalization or death, as identified from the VHA Corporate Data Warehouse (CDW). Death dates were recorded in the patient vital status table and hospitalizations were recorded in the inpatient table. Patients were followed from the index date until the outcome of interest occurred or the censoring date due to magnesium supplement use status change (i.e., stop use in users or start use in non-users), prescribed magnesium replacement was started, or the end of 5th year since the index date, whichever occurred first. To account for potential differences in follow-up time since magnesium supplement non-users are more likely to maintain the same status than users, a magnesium supplement user and their paired non-user with the same index date were censored at the same date if either side were censored.

### 2.6. Study Covariates

We included an extensive list of baseline variables as covariates aimed at controlling for potential confounding and to ensure comparability between the two treatment strategy groups. Baseline covariates included demographics, socio-environmental and economic status, duration between the initial HF diagnosis and the index date, comorbid conditions, medication use, laboratory data, vital signs, and healthcare utilization.

Median household income and the socio-environmental percentile ranking were estimated at the patients’ residential address zip code. The socio-environmental percentile Environmental Justice Index (EJI) developed by the Center for Disease Control (CDC) was used to measure the cumulative impact of environmental burden on human health and the environment at the patients’ residence. A higher percentile EJI ranking indicates a greater cumulative socio-environmental burden on the community [19]. Comorbid conditions, identified using ICD codes, were captured any time before or at the index date. Other covariates were captured within one year prior to and up to the index date, with the measures closest to the index date used as baseline data, which included medication use from prescription/fill records, laboratory tests using LOINC codes, vital signs from vital sign records, and healthcare utilization from outpatient and inpatient encounter records.

### 2.7. Variables with Missing Values

For vital signs and laboratory values, if the missing value rate was less than 30%, we imputed the values using the mean, conditional on age, gender, race, and ethnicity; otherwise, we converted the variables into categorical ones using missing as one of the possible categories to adjust for potential bias from informative missing. The following variables were imputed, with missing rates (magnesium supplement users vs. non-users) as follows: 4.7% (5.4% vs. 4.1%) for body mass index (BMI), 2.5% (2.9% vs. 2.2%) for systolic blood pressure, 2.6% (3.0% vs. 2.2%) for diastolic blood pressure, 23.4% (26.5% vs. 20.3%) for low-density lipoprotein, 23.9% (27.2% vs. 20.6%) for triglycerides, 22.7% (25.9% vs. 19.5%) for high-density lipoprotein, 22.4% (25.6% vs. 19.2%) for total cholesterol, 13.7% (18.0% vs. 9.3%) for serum sodium, 13.6% (17.9% vs. 9.4%) for serum potassium, 25.6% (28.4% vs. 22.8%) for serum creatinine, 18.4% (22.9% vs. 13.9%) for serum calcium, and 29.7% (32.3% vs. 27.0%) for hemoglobin A1C. This indicates that magnesium supplement users had a slightly higher rate of missing values.

### 2.8. Statistical Analysis

We used the per-protocol approach for data analysis as described earlier, and patients were censored when they changed the treatment strategy.

We fit a logistic regression model to estimate the propensity score (i.e., the probability of being a magnesium supplement user) using baseline characteristics including demographics, comorbid conditions, concurrent medications, health care utilization in the past year, vital signs and labs. Then, we applied inverse probability treatment weighting (IPTW) to generate a pseudo-population, in which baseline characteristics of two treatment strategy groups were balanced (ASD < 10%). Stabilized weights (SW) were calculated for each patient. To avoid extreme values, when a weight was above 10, it was trimmed down to 10 [20]. The absolute standardized difference (ASD) for each characteristic was calculated before and after weighting, with an ASD > 10% indicating a significant imbalance in characteristics between the two groups [21].

We used the inverse probability of treatment (IPT)-weighted cohort to estimate the outcome incidence rates of the two groups. Kaplan–Meier curves were plotted, and Cox’s regression modeling was applied to the weighted cohort to assess the association between magnesium supplement use and the risk of all-cause hospitalization or death. The proportional hazards assumption was tested by including an interaction term between magnesium supplement use and time in the Cox’s regression model to examine any time-dependent effects on outcomes.

Additionally, we evaluated statistical interactions between magnesium supplement use and potential effect modifiers, including age, race, serum magnesium level, serum vitamin D level, vitamin D prescription, multivitamin use, and diuretic use, by incorporating a product term in the regression models. When a product term was statistically significant, subgroup analyses were conducted. In these subgroup analyses, we repeated the IPTW process to assess the association between magnesium supplement use and adverse outcomes within each subgroup.

### 2.9. Sensitivity Analyses

We also conducted two sensitivity analyses. In the first sensitivity analysis, we restricted the cohort to the subgroup who did not die within 6 months after the index date to exclude a very ill population unlikely to have benefited from magnesium supplement use. In the second sensitivity analysis, we restricted the cohort to the subgroup who had at least one additional clinical note mentioning magnesium supplement use and their paired controls to minimize misclassification bias. For each sensitivity analysis, we recalculated the IPTW to make magnesium supplement users and non-users comparable.

All analyses were performed using SAS (Version 9.4). A two-sided *p*-value of <0.05 was considered statistically significant.

### 2.10. Target Trial Assumption Evaluation

Consistency: We performed sensitivity analyses mentioned above that assessed the robustness of the results to potential violations of consistency, addressing issues such as exposure-related missing data, time-varying confounding, or measurement error.

Positivity: We evaluated the probability of being exposed or unexposed to magnesium supplement use within each subgroup, based on baseline characteristics, to ensure that observations existed for both exposure status in every subgroup. The positivity assumption was satisfied in this study.

Exchangeability: Although there is always a possibility of unknown confounding, we included an expansive number of variables mentioned above to control for potential confounding bias.

## 3. Results

### 3.1. Baseline Characteristics

Between 1 January 2000, and 31 December 2020, a total of 1,194,095 veterans received a first-time diagnosis of HF within the VHA system. For the target trial emulation, we enrolled all 9900 individuals who met eligibility criteria and used magnesium supplements and matched them to 9900 randomly selected non-users who had a clinic visit on the same index date. Patient characteristics before and after weighting are presented in Table 2 and Table 3 and Supplementary Figure S1.

Prior to weighting, many baseline characteristics were unbalanced (ASD > 10%) between treatment groups. Magnesium supplement users were older (73.3 vs. 72 years) and less likely to be African American (8.8% vs. 15.7%) than non-users. They also had a more favorable socio-environmental percentile ranking (46.3 vs. 49.6) and a shorter average interval between HF diagnosis and the index date (105.6 days vs. 153.7 days). Magnesium users were less likely than non-users to have a history of alcohol abuse (12.0% vs. 16.5%) or smoking (25.1% vs. 36.4%). They had lower rates of hypertension (84.2% vs. 89.7%), hyperlipidemia (79.3% vs. 83.1%), myocardial infarction (15.5% vs. 20.5%), angina (13.6% vs. 20.7%), and other comorbid conditions, including history of chronic obstructive pulmonary disease (COPD), anemia, arthritis, cancer, urinary tract infection (UTI), pneumonia, respiratory failure, sepsis, depression, and fracture. However, they were more likely to have atrial fibrillation (36.8% vs. 30.9%) and low serum magnesium levels (7.6% vs. 2.5%) at baseline. In addition, magnesium supplement users were less likely to have a known left-ventricular ejection fraction (43.6% vs. 32.6% unknown) and were less likely to be treated with medications including insulin, antihypertensives, cholesterol medications, aspirin, glucocorticoids, and platelet inhibitors. The prevalence of left-ventricular ejection fraction ≤ 40% was similar between the groups (26.9% vs. 25.4%).

**Figure 3:**
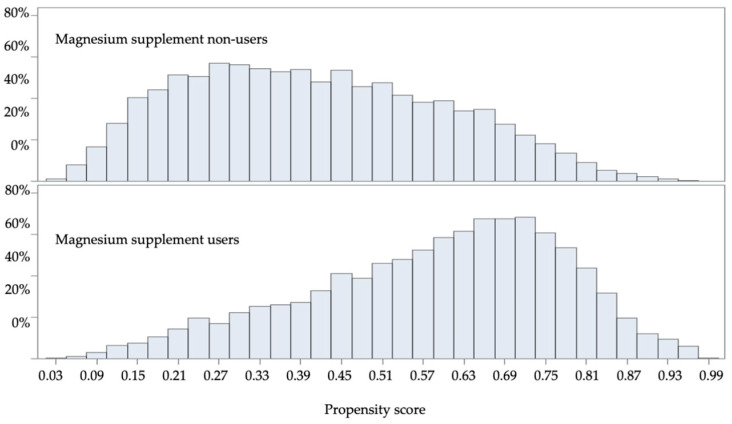
Propensity score distribution.

The propensity score distributions of magnesium supplement users and non-users are shown in Figure 3, indicating adequate overlap and no violation of the positivity assumption. The mean and standard deviation of IPTWs were 1.01 ± 0.58 overall (0.99 ± 0.52 for non-magnesium users and 1.02 ± 0.62 for magnesium supplement users). The ASD values for all measured baseline characteristics in the weighted cohort were below 10%, indicating balance after weighting (Table 3). The IPT-weighted pseudo-cohort had a mean age of 72.6 ± 11.1 years and comprised 12.6% African Americans, 3.3% Hispanics, and 3.4% women. Among these patients in the pseudo-cohort, 87.2% had hypertension, 33.9% had atrial fibrillation, 18.4% had myocardial infarction, and 12.7% had ischemic stroke. Furthermore, 14.5% and 31.5% of patients had a history of alcohol abuse and smoking, respectively. Overall, 39.8% of patients were treated with anti-diabetes medications and 77.1% with anti-hypertensive medications.

### 3.2. Magnesium Supplement Use and Adverse Outcomes

#### 3.2.1. Descriptive Analysis

The IPT-weighted cohort was followed for up to 5 years (median follow-up: 0.7 years for magnesium supplement non-users and 0.6 years for magnesium supplement users), with a mean (±standard deviation [SD]) follow-up duration of 0.85 ± 0.96 years for magnesium supplement users and 0.82 ± 0.95 years for non-users.

Among the 9900 magnesium supplement users, 3532 (35.7%) had no follow-up clinical note referencing magnesium supplement use after the eligible date; for these patients, magnesium supplementation was considered discontinued one year after the last recorded clinical note. For the remaining 6368 (64.3%) magnesium users, the mean duration of magnesium supplement use was 1.9 ± 2.0 years (median: 1.1 years; interquartile range [IQR]: 0.6–2.6 years) without accounting for the timing of outcomes or censoring events and 0.8 ± 1.0 years (median 0.4 years; IQR: 0.1–1.0 years) when accounting for the occurrence of an outcome or a censoring event.

The incidence of all-cause hospitalization or death was 24.3% (297.8 events per 1000 person-years) among magnesium supplement users and 30.7% (360.2 events per 1000 person-years) among non-users in the weighted cohort (Table 4).

#### 3.2.2. Main and Sensitivity Analyses

**Figure 4:**
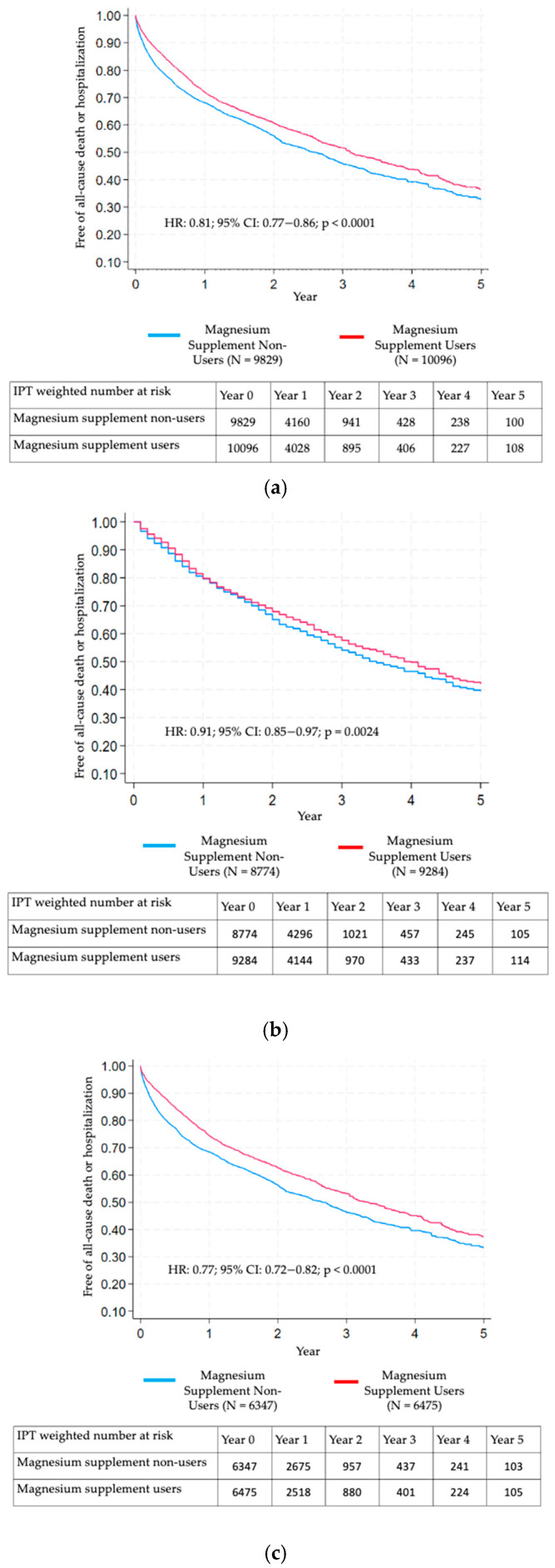
(**a**) KM Curves of all-cause death or hospitalization comparing inverse probability of treatment-weighted magnesium supplement users vs. non-users. (**b**) KM curves of all-cause death or hospitalization comparing inverse probability of treatment-weighted magnesium supplement users vs. non-users (sensitivity analysis in the subgroup who did not die within 6 months after the index date). (**c**) KM curves of all-cause death or hospitalization comparing inverse probability of treatment-weighted magnesium supplement users vs. non-users (sensitivity analysis in the subgroup who had at least one additional clinical note mentioning magnesium supplement use and their paired controls).

The weighted cohort showed that magnesium users had a significantly higher death-or-hospitalization-free survival compared to non-users (*p* < 0.0001 for the log-rank test), as shown in Figure 4a. Cox’s regression modeling indicated that magnesium supplement use was associated with a significantly reduced risk of incident all-cause hospitalization or death (HR: 0.81; 95% CI: 0.77–0.86; *p* < 0.0001).

Similar associations were observed in the sensitivity analyses of the subgroup who survived at least 6 months (n = 17,959; HR: 0.91; 95% CI: 0.85–0.97; *p* = 0.0024, Figure 4b) from the index date and in the restricted cohort who had at least one more clinic visit note during follow-up confirming sustained magnesium supplement use along with their paired controls (n = 12,736; HR: 0.77; 95% CI: 0.72–0.82; *p* < 0.0001, Figure 4c).

#### 3.2.3. Interactions and Stratified Subgroup Analyses

The interaction between magnesium supplement use and follow-up time was not statistically significant (*p* = 0.7689), indicating that the proportional hazard assumption was not violated. The association between magnesium supplement use and a reduced risk of all-cause death or hospitalization was homogeneous, with no significant interactions, across subgroups of the IPTW-weighted cohort stratified by gender (*p* = 0.8413), BMI (*p* = 0.1875), serum vitamin D levels (*p* = 0.1469), prescribed multivitamin (*p* = 0.9108), diuretics (*p* = 0.1114), serum magnesium level (*p* = 0.7719), and left-ventricular ejection fraction (*p* = 0.1648).

**Figure 5:**
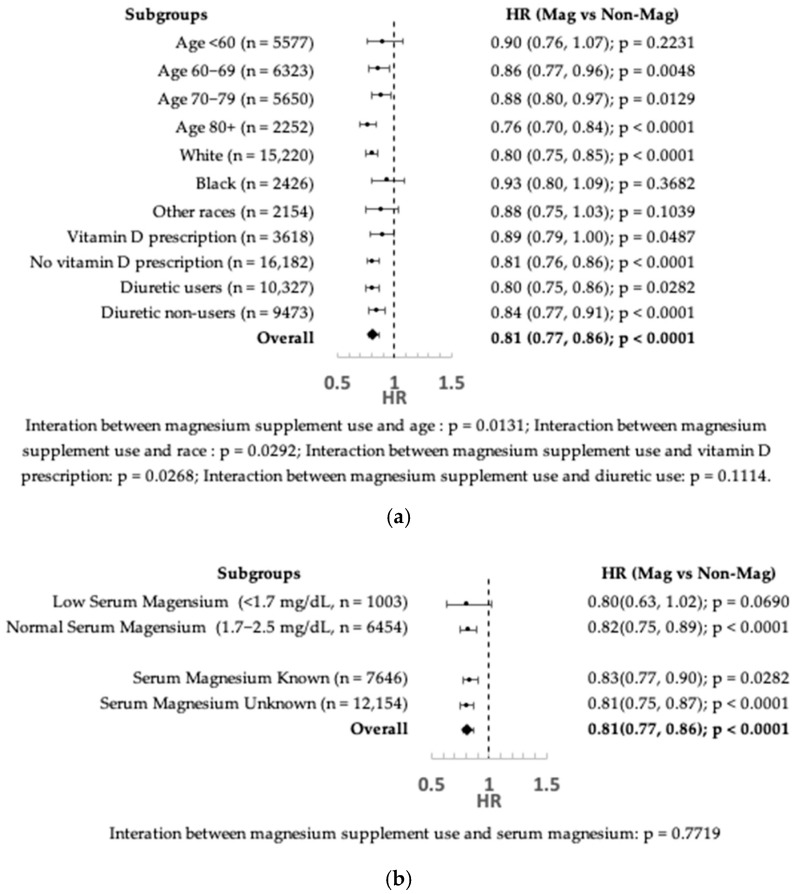
(**a**) Subgroup analysis by variables with significant interactions. (**b**) Subgroup analysis by serum magnesium.

The association between magnesium supplement use and reduced outcome risk appeared stronger in older individuals than younger ones (*p* = 0.0131 for interaction with age) and in the European American population than other racial subgroups (*p* = 0.0292 for interaction with race) (Figure 5a). Vitamin D prescription also significantly modified the association between magnesium supplement use and adverse outcomes, with an additional 8% risk reduction in adverse outcome in patients who were not prescribed with vitamin D compared to those who were prescribed (*p* = 0.0268 for interaction with vitamin D prescription). Although diuretics did not have a differential effect on the association between magnesium supplementation and the outcomes, a slightly stronger effect size for magnesium supplement use was observed in diuretic users than in non-users.

Furthermore, no heterogeneity was observed in the association between magnesium supplement use and the outcomes when stratified by knowledge of serum magnesium levels (Figure 5b).

## 4. Discussion

Our study found that patients with documented use of magnesium supplements within one year after diagnosed HF were associated with a 19% reduction in the risk of all-cause mortality or hospitalization compared to those without any recorded magnesium supplement use. The effect size was even greater among individuals with sustained use, showing a 23% relative risk reduction. However, when the analysis was limited to patients who survived at least six months from the index date, the effect size was less pronounced, with the KM curves beginning to diverge at around two years after initiation as opposed to within the first year.

Magnesium is essential for maintaining normal heart rhythm [22] and plays a critical role in the prevention of atrial fibrillation [23] and other types of cardiac arrhythmia [24]. Although many studies have shown that magnesium replacement is linked to a reduced risk of adverse outcomes related to HF [25,26,27], there is lack of research on the effects of magnesium supplementation. Only a few studies related to dietary magnesium have been reported. A reverse association between dietary magnesium and the 10-year risk of atherosclerotic cardiovascular disease events was observed in a cross-sectional study conducted among 2980 participants aged 40–70 years, based on data from the National Health and Nutrition Examination Survey (1999–2018) [28]. Another observational study found that magnesium intake was significantly associated with a reduction in 28-day all-cause mortality (HR: 0.68; 95% CI: 0.54–0.86) among 1970 patients with HF with preserved ejection fraction (HFpEF) who had at least one admission to the intensive care unit (ICU) [29]. Our findings expand the prior studies to examine magnesium supplement use in a large cohort of veteran patients with various types of HF, with a follow-up period of up to 5 years.

These finding have important implications, given that approximately 6 million Americans are currently living with HF [30]. Dietary supplements, such as magnesium, are relatively low-cost and generally considered safe. The results suggest that magnesium supplementation in patients with HF who do not have end-stage renal disease may offer potential health benefits. The observed benefits were more pronounced in older individuals, particularly those aged 80 years or older. Since age-related declines in magnesium absorption and the use of some medications in the elderly may reduce magnesium levels [31,32], our findings add evidence to support dietary magnesium supplementation in patients at risk of magnesium deficit. Regarding the heterogeneity of magnesium supplements by racial groups, it is likely related to variations in average dietary intake of magnesium and prevalence of certain chronic health conditions rather than a difference in how magnesium works in the body. Given the low sample size of non-white races in the study cohort, further investigation is needed.

Vitamin D and magnesium interact closely: vitamin D cannot function effectively when magnesium levels are low [33]. It is likely that individuals prescribed with vitamin D have low vitamin D levels, which may alter magnesium distribution and utilization. This may explain why magnesium supplementation had a stronger effect size in patients without a vitamin D prescription, presumably due to likely normal vitamin D levels.

Since diuretics may cause hypomagnesemia [34,35], we conducted a subgroup analysis stratified by diuretic use and observed slight heterogeneity, with more beneficial effects in diuretic users than in non-users. This finding suggests that magnesium supplementation should be recommended for diuretic users, as supported by the literature [36].

Our study has limitations. First, magnesium supplement use was identified through clinician-documented history rather than prescription data. This raises the possibility that some patients may have begun using magnesium supplements before their first reported use, which would reflect prevalent rather than incident use. In addition, data on adherence to magnesium supplements would also have the same misclassification bias. To minimize this bias, we used continuously documented use to document adherence. There is a likelihood of misclassifying the treatment strategies (i.e., magnesium users as non-users and vice versa due to self-report or documentation deficits), which may bias our estimates towards the null. We also performed sensitivity analyses to assess the robustness of our results, which remained consistent. Also, the lack of a unified formulation or dose of magnesium supplements raises the question of what the ideal amount of elemental magnesium supplementation is and whether a dose–response relationship exists. According to the literature, the most commonly used formulations for magnesium supplements in the US (magnesium glycinate, citrate, and oxide) contain a range of elemental magnesium from 27–242 mg [37], most of which attempt to match the Recommended Dietary Allowance (RDA) dose for adult men and women (400–420 mg and 310–360 mg, respectively) [38].

Although we adjusted for a wide range of potential confounders, including social and environmental factors, to generate IPTWs, residual confounding may still be present, such as dietary and exercise factors. The social and environmental variables, including residential area median income and the EJI socio-environmental percentile ranking, were included to account for differences in health outcomes that may be influenced by healthier diets, lifestyles, and the activeness of health-seeking behaviors. Conversely, the study population was considered a high-risk and hard-to-recruit population in the real life, as reflected by the high number of adverse events occurring within the first six months. However, the sensitivity analysis restricted to patients who survived at least six months showed consistent results and suggests that the results can also be generalizable to lower-risk patients with HF. Lastly, the generalizability of our findings to women with HF or community-based HF patients may be limited due to the predominantly elderly male veteran population included in the study. Despite this limitation, this study was still able to include 667 women. We chose veterans as the study population because the VHA databases contain comprehensive variables related to health history and records—such as comorbidities, laboratory results, pharmacy data, health care utilization, and vital signs—which may help address potential confounding and indication biases associated with both magnesium supplement use and the outcome. Future studies need to be conducted in other populations.

## 5. Conclusions

In conclusion, our findings indicate that magnesium supplementation is linked to a lower risk of all-cause mortality or hospitalization in patients with HF. These promising outcomes merit further exploration and validation through RCTs.

## Figures and Tables

**Table 1:** Elements of the target trial and emulation for magnesium supplement treatment strategies.

	Target Trial	Emulation in Observational Data
Aim	Evaluate the association between magnesium supplement use and all-cause hospitalization or death in the U.S. military veterans with HF	
Eligibility	(1) Newly diagnosed with HF no more than 1 year (2) Never used magnesium supplement or replacement treatment before (3) No hospitalization in the past 30 days (4) No end-stage renal disease (ESRD)	(1) Had the initial HF diagnosis within 1 year before (2) Had at least two outpatient clinical notes within the previous 12 months (to confirm magnesium supplement use status) (3) Had no record of prescribed or supplemental magnesium use prior to this date (4) Had no hospitalization within 30 days before or at the eligible date (5) Had no ESRD any time before or at the eligible date
Treatment Strategies	(1) Magnesium supplement intervention: Initiation of magnesium supplement (2) Magnesium supplement non-intervention: No initiation of magnesium supplement	(1) Magnesium supplement users: new documentation of the use of magnesium supplements in an outpatient clinic encounter (2) Magnesium supplement non-users: No documentation of magnesium supplement use in an outpatient clinic encounter
Assignment	Randomly assigned 1:1 to each treatment strategy in a parallel design	Veterans were assigned to treatment arms based on EMR documentation. All magnesium supplement users were included. For the non-user group, randomization was emulated by randomly selecting a group of non-user patients with the same eligible date as a user. Inverse probability weighting was used to balance observed confounders between treatment groups.
Follow-up	From date of randomization up until end of 5 years, outcome, or censoring due to events such as violation of the assigned treatment strategy	Followed from the time when a patient became eligible for the trial (index date) until the outcome of interest occurred, or the censoring date due to magnesium supplement use status change (i.e., stop use in users or start use in non-users), prescribed magnesium replacement was started, or the end of 5th year from the index date, whichever occurred first.
Outcomes	All-cause mortality or all-cause hospitalization (time to events)	Same
Causal Contrast	The difference in risk of all-cause mortality or all-cause hospitalization had everyone followed strategy (1) vs. strategy (2)	Same
Statistical Analysis	Cox’s regression	Per-protocol analysis using Cox’s regression weighted by inverse probability of treatment

**Table 2:** Baseline characteristics of study participants before IPT weighting.

Baseline Characteristics	Overall (N = 19,800)		Magnesium Users (N = 9900)		Magnesium Non-Users (N = 9900)		ASD Before IPTW (%)
	Mean/N	SD/%	Mean/N	SD/%	Mean/N	SD/%	
Demographics (8 Variables)							
Age, Years	72.6	11.1	73.3	10.7	72.0	11.4	12
Male	19,133	96.6%	9544	96.4%	9589	96.9%	3
Race							
White	15,220	76.9%	7872	79.5%	7348	74.2%	13
Black	2426	12.3%	869	8.8%	1557	15.7%	21
Others	2154	10.9%	1159	11.7%	995	10.1%	5
Ethnicity							
Non-Hispanics	17,965	90.7%	8991	90.8%	8974	90.6%	1
Hispanics	640	3.2%	244	2.5%	396	4.0%	8
Unknown	1195	6.0%	665	6.7%	530	5.4%	5
Married	11,583	58.5%	6262	63.3%	5321	53.7%	20
Median Income (lowest to highest) of Zip Code							
1st Quartile	4128	20.8%	1914	19.3%	2214	22.4%	8
2nd Quartile	4811	24.3%	2478	25.0%	2333	23.6%	3
3rd Quartile	4871	24.6%	2472	25.0%	2399	24.2%	2
4th Quartile	5530	27.9%	2865	28.9%	2665	26.9%	4
Unknown	460	2.3%	171	1.7%	289	2.9%	8
Environmental Justice Index Socio-Environmental Percentile Ranking of Zip code	48.0	22.6	46.3	22.2	49.6	22.9	15
Duration between HF Diagnosis and Index Date, Days	129.6	117.7	105.6	118.1	153.7	112.2	42
Comorbid Conditions (31 Variables)							
Alcohol Disorder	2818	14.2%	1188	12.0%	1630	16.5%	13
Smoking	6083	30.7%	2483	25.1%	3600	36.4%	25
Hypertension	17,218	87.0%	8340	84.2%	8878	89.7%	16
Hyperlipidemia	16,079	81.2%	7849	79.3%	8230	83.1%	10
Myocardial Infarction	3566	18.0%	1536	15.5%	2030	20.5%	13
Atherosclerosis	4770	24.1%	2208	22.3%	2562	25.9%	8
Angina	3394	17.1%	1346	13.6%	2048	20.7%	19
Atrial Fibrillation	6705	33.9%	3646	36.8%	3059	30.9%	12
Cardiac Valve Disease	3430	17.3%	1574	15.9%	1856	18.7%	7
Ischemic Stroke	2460	12.4%	1083	10.9%	1377	13.9%	9
Hemorrhage Stroke	172	0.9%	62	0.6%	110	1.1%	5
Transient Ischemic Attack	1061	5.4%	429	4.3%	632	6.4%	9
Chronic Obstructive Pulmonary Disease	7125	36.0%	3324	33.6%	3801	38.4%	10
Asthma	1820	9.2%	856	8.6%	964	9.7%	4
Chronic Kidney Disease	5612	28.3%	2672	27.0%	2940	29.7%	6
Anemia	6541	33.0%	3008	30.4%	3533	35.7%	11
Arthritis	9347	47.2%	4398	44.4%	4949	50.0%	11
Cancer	6753	34.1%	3129	31.6%	3624	36.6%	11
Neurological Disorders	7556	38.2%	3572	36.1%	3984	40.2%	8
Parkinson	417	2.1%	181	1.8%	236	2.4%	4
Hypothyroidism	2887	14.6%	1511	15.3%	1376	13.9%	4
Osteoporosis	866	4.4%	420	4.2%	446	4.5%	1
Osteomyelitis	500	2.5%	183	1.8%	317	3.2%	9
Urinary Tract Infection	1238	6.3%	487	4.9%	751	7.6%	11
Liver Disease	1875	9.5%	828	8.4%	1047	10.6%	8
Pneumonia	2481	12.5%	917	9.3%	1564	15.8%	20
Respiratory Failure	1087	5.5%	373	3.8%	714	7.2%	15
Sepsis	662	3.3%	210	2.1%	452	4.6%	14
Opioid Use Disorder	420	2.1%	150	1.5%	270	2.7%	8
Depression	6883	34.8%	2998	30.3%	3885	39.2%	19
Fracture	2223	11.2%	915	9.2%	1308	13.2%	13
Concurrent Medications (22 Variables)							
Insulin	5041	25.5%	2214	22.4%	2827	28.6%	14
Metformin	3949	19.9%	1855	18.7%	2094	21.2%	6
Glucagon-Like Peptide-1	78	0.4%	34	0.3%	44	0.4%	2
Sodium–Glucose Cotrasporter-2 Inhibitors	195	1.0%	100	1.0%	95	1.0%	0
Other Diabetes Medication	3221	16.3%	1470	14.8%	1751	17.7%	8
Vitamin D Prescription	3618	18.3%	1433	14.5%	2185	22.1%	20
Multivitamin (Rx)	972	4.9%	390	3.9%	582	5.9%	9
Angiotensin-Converting Enzyme Inhibitor	8021	40.5%	3229	32.6%	4792	48.4%	33
Angiotensin Receptor Blocker	3137	15.8%	1446	14.6%	1691	17.1%	7
Calcium Channel Blocker	5442	27.5%	2211	22.3%	3231	32.6%	23
Loop Diuretics	8765	44.3%	3750	37.9%	5015	50.7%	26
Thiazide Diuretics	3262	16.5%	1372	13.9%	1890	19.1%	14
Selective Beta Blocker	7996	40.4%	3287	33.2%	4709	47.6%	30
Non-Selective Beta Blocker	4425	22.3%	1913	19.3%	2512	25.4%	15
Other Antihypertensive	5931	30.0%	2598	26.2%	3333	33.7%	16
Statins	11,125	56.2%	4679	47.3%	6446	65.1%	36
Other Lipid-Lowering Medication	1258	6.4%	573	5.8%	685	6.9%	5
Proton-Pump Inhibitors	6662	33.6%	2940	29.7%	3722	37.6%	17
Aspirin	5894	29.8%	2067	20.9%	3827	38.7%	40
Digoxin	334	1.7%	154	1.6%	180	1.8%	2
Glucocorticoids	2443	12.3%	972	9.8%	1471	14.9%	16
Platelet Inhibitor	3058	15.4%	1280	12.9%	1778	18.0%	14
Health Care Utilization (11 Variables)							
Hospitalization(s) in past year							
0	14,860	75.1%	8364	84.5%	6496	65.6%	45
1	3035	15.3%	976	9.9%	2059	20.8%	31
2+	1905	9.6%	560	5.7%	1345	13.6%	27
Number of visits in past year	31.5	29.5	26.8	28.0	36.2	30.2	32
Addiction Medicine	483	2.4%	186	1.9%	297	3.0%	7
Cardiology	8551	43.2%	3170	32.0%	5381	54.4%	46
Emergency Care	5800	29.3%	2025	20.5%	3775	38.1%	39
Endocrinology	990	5.0%	425	4.3%	565	5.7%	6
Hospice Medicine	138	0.7%	54	0.5%	84	0.8%	4
Intensive Care Unit	1255	6.3%	379	3.8%	876	8.8%	21
Internal Medicine	6184	31.2%	2628	26.5%	3556	35.9%	20
Oncology	980	4.9%	404	4.1%	576	5.8%	8
Palliative Care	338	1.7%	115	1.2%	223	2.3%	8
Vital Signs and Labs (14 Variables)							
Body Mass Index, kg/m^2^	30.0	6.9	30.2	6.9	29.8	6.9	6
Systolic Blood Pressure, mmHg	129.5	19.9	127.9	19.8	131.0	19.9	16
Diastolic Blood Pressure, mmHg	72.1	11.7	71.3	11.5	72.9	11.9	14
Serum Magnesium, mg/dL							
<1.7 mg/dL	1003	5.1%	757	7.6%	246	2.5%	23
1.7–2.5 mg/dL	6454	32.6%	2608	26.3%	3846	38.8%	27
>2.5 mg/dL	189	1.0%	90	0.9%	99	1.0%	1
Unknown	12,154	61.4%	6445	65.1%	5709	57.7%	15
Serum 25-hydroxy vitamin D < 20 ng/dL	927	4.7%	382	3.9%	545	5.5%	8
Low-Density Lipoprotein, mg/dL	85.9	34.2	85.1	34.5	86.5	33.9	4
Triglycerides, mg/dL	146.3	111.5	147.1	110.3	145.6	112.6	1
High-Density Lipoprotein, mg/dL	43.2	14.2	43.3	14.4	43.0	14.0	2
Cholesterol, mg/dL	155.3	42.6	154.5	42.9	156.0	42.3	4
Sodium, mmol/L	138.9	3.2	138.7	3.3	139.0	3.1	9
Potassium, mmol/L	4.3	0.5	4.3	0.5	4.3	0.5	0
Creatinine, mg/dL	1.3	0.6	1.3	0.6	1.3	0.6	0
Calcium, mg/dL	9.2	0.5	9.2	0.5	9.2	0.5	0
Hemoglobin A1C, %	6.8	1.5	6.9	1.5	6.8	1.5	7
Ejection Fraction							
≤40%	5171	26.1%	2600	26.9%	2511	25.4%	3
>40%	7091	35.8%	2928	29.6%	4163	42.1%	26
Unknown	7538	38.1%	4312	43.6%	3226	32.6%	23

Abbreviations: SD: standard deviation; ASD: absolute standardized difference; IPTW: inverse probability of treatment weighting.

**Table 3:** Baseline characteristics of study participants after IPT weighting.

Baseline Characteristics	IPT-Weighted Overall (N = 19,925)		IPT-Weighted Magnesium Users (N = 10,096)		IPT-Weighted Magnesium Non-Users (N = 9829)		ASD After IPTW (%)
	Mean/N	SD/%	Mean/N	SD/%	Mean/N	SD/%	
Demographics (8 Variables)							
Age, Years	72.6	11.1	72.5	10.8	72.6	11.4	1
Male	19,240	96.6%	9748	96.6%	9492	96.6%	0
Race							
White	15,260	76.6%	7711	76.4%	7550	76.8%	1
Black	2518	12.6%	1300	12.9%	1219	12.4%	2
Others	2147	10.8%	1086	10.8%	1061	10.8%	0
Ethnicity							
Non-Hispanics	18,105	90.9%	9171	90.8%	8935	90.9%	0
Hispanics	654	3.3%	336	3.3%	318	3.2%	1
Unknown	1166	5.9%	589	5.8%	577	5.9%	0
Married	11,565	58.0%	5828	57.7%	5737	58.4%	1
Median Income (lowest to highest) of Zip Code							
1st Quartile	4197	21.1%	2139	21.2%	2058	20.9%	1
2nd Quartile	4797	24.1%	2424	24.0%	2373	24.1%	0
3rd Quartile	4825	24.2%	2438	24.1%	2387	24.3%	0
4th Quartile	5624	28.2%	2842	28.1%	2782	28.3%	0
Unknown	482	2.4%	253	2.5%	229	2.3%	1
Environmental Justice Index Socio-Environmental Percentile Ranking of Zip code	47.9	22.7	48.0	22.9	47.8	22.6	1
Duration between Diabetes Diagnosis and Index Date, Days	135.0	118.4	136.0	125.8	134.0	110.5	2
Comorbid Conditions (31 Variables)							
Alcohol Disorder	2888	14.5%	1471	14.6%	1418	14.4%	1
Smoking	6267	31.5%	3210	31.8%	3058	31.1%	2
Hypertension	17,382	87.2%	8804	87.2%	8578	87.3%	0
Myocardial Infarction	3670	18.4%	1866	18.5%	1804	18.4%	0
Atherosclerosis	4884	24.5%	2478	24.5%	2405	24.5%	0
Angina	3548	17.8%	1817	18.0%	1731	17.6%	1
Atrial Fibrillation	6756	33.9%	3424	33.9%	3331	33.9%	0
Cardiac Valve Disease	3565	17.9%	1824	18.1%	1741	17.7%	1
Ischemic Stroke	2530	12.7%	1284	12.7%	1246	12.7%	0
Hemorrhage Stroke	185	0.9%	97	1.0%	88	0.9%	1
Transient Ischemic Attack	1092	5.5%	552	5.5%	539	5.5%	0
Chronic Obstructive Pulmonary Disease	7263	36.5%	3703	36.7%	3560	36.2%	1
Asthma	1922	9.6%	989	9.8%	934	9.5%	1
Chronic Kidney Disease	5769	29.0%	2919	28.9%	2850	29.0%	0
Anemia	6767	34.0%	3452	34.2%	3314	33.7%	1
Arthritis	9532	47.8%	4820	47.7%	4712	47.9%	0
Cancer	6905	34.7%	3488	34.6%	3417	34.8%	0
Neurological Disorders	7785	39.1%	3953	39.1%	3832	39.0%	0
Parkinson	421	2.1%	218	2.2%	204	2.1%	1
Hypothyroidism	2944	14.8%	1498	14.8%	1446	14.7%	0
Osteoporosis	888	4.5%	459	4.6%	428	4.4%	1
Osteomyelitis	521	2.6%	268	2.7%	254	2.6%	1
Hyperlipidemia	16,306	81.8%	8244	81.7%	8062	82.0%	1
Urinary Tract Infection	1344	6.7%	711	7.0%	633	6.4%	2
Liver Disease	1971	9.9%	1006	10.0%	965	9.8%	1
Pneumonia	2592	13.0%	1341	13.3%	1251	12.7%	2
Respiratory Failure	1175	5.9%	622	6.2%	553	5.6%	3
Sepsis	699	3.5%	362	3.6%	338	3.4%	1
Opioid Use Disorder	437	2.2%	229	2.3%	208	2.1%	1
Depression	7050	35.4%	3595	35.6%	3454	35.1%	1
Fracture	2378	11.9%	1235	12.2%	1144	11.6%	2
Concurrent Medications (22 Variables)							
Insulin	5219	26.2%	2656	26.3%	2564	26.1%	0
Metformin	4099	20.6%	2049	20.3%	2051	20.9%	1
Glucagon-Like Peptide-1	84	0.4%	42	0.4%	42	0.4%	0
Sodium–Glucose Cotrasporter-2 Inhibitors	208	1.0%	106	1.0%	103	1.0%	0
Other Diabetes Medication	3315	16.6%	1663	16.5%	1652	16.8%	1
Vitamin D Prescription	3789	19.0%	1941	19.2%	1848	18.8%	1
Multivitamin (Rx)	1044	5.2%	536	5.3%	507	5.2%	0
Angiotensin-Converting Enzyme Inhibitor	8280	41.6%	4227	41.9%	4053	41.2%	1
Angiotensin Receptor Blocker	3253	16.3%	1633	16.2%	1620	16.5%	1
Calcium Channel Blocker	5663	28.4%	2892	28.6%	2771	28.2%	1
Loop Diuretics	9176	46.1%	4666	46.2%	4510	45.9%	1
Thiazide Diuretics	3339	16.8%	1683	16.7%	1657	16.9%	1
Selective Beta Blocker	8278	41.5%	4202	41.6%	4077	41.5%	0
Non-Selective Beta Blocker	4636	23.3%	2362	23.4%	2274	23.1%	1
Other Antihypertensive	6195	31.1%	3143	31.1%	3052	31.1%	0
Statins	11,391	57.2%	5788	57.3%	5603	57.0%	1
Other Lipid-Lowering Medication	1276	6.4%	630	6.2%	646	6.6%	2
Proton-Pump Inhibitors	6903	34.6%	3487	34.5%	3415	34.7%	0
Aspirin	6180	31.0%	3181	31.5%	2999	30.5%	2
Digoxin	332	1.7%	164	1.6%	168	1.7%	1
Glucocorticoids	2584	13.0%	1320	13.1%	1264	12.9%	1
Platelet Inhibitor	3171	15.9%	1605	15.9%	1567	15.9%	0
Health Care Utilization (11 Variables)							
Hospitalization(s) in past year							
0	14,672	73.6%	7372	73.0%	7300	74.3%	3
1	3217	16.1%	1657	16.4%	1561	15.9%	1
2+	2036	10.2%	1068	10.6%	968	9.9%	2
Number of visits in past year	32.8	30.3	33.1	31.6	32.6	29.0	2
Addiction Medicine	491	2.5%	251	2.5%	240	2.4%	1
Cardiology	8874	44.5%	4550	45.1%	4324	44.0%	2
Emergency Care	6134	30.8%	3146	31.2%	2988	30.4%	2
Endocrinology	1058	5.3%	529	5.2%	529	5.4%	1
Hospice Medicine	164	0.8%	92	0.9%	71	0.7%	2
Intensive Care Unit	1322	6.6%	684	6.8%	638	6.5%	1
Internal Medicine	6416	32.2%	3293	32.6%	3123	31.8%	2
Oncology	1052	5.3%	548	5.4%	504	5.1%	1
Palliative Care	389	2.0%	213	2.1%	175	1.8%	2
Vital Signs and Labs (14 Variables)							
Body Mass Index, kg/m^2^	30.0	6.9	29.9	7.0	30.0	6.8	1
Systolic Blood Pressure, mmHg	129.7	20.2	129.8	20.9	129.6	19.6	1
Diastolic Blood Pressure, mmHg	72.3	12.0	72.4	12.2	72.2	11.7	2
Serum Magnesium, mg/dL							
<1.7 mg/dL	1070	5.4%	511	5.1%	559	5.7%	3
1.7–2.5 mg/dL	6711	33.7%	3448	34.2%	3263	33.2%	2
>2.5 mg/dL	190	1.0%	95	0.9%	95	1.0%	1
Unknown	11,954	60.0%	6042	59.8%	5912	60.2%	1
Serum 25-hydroxy vitamin D < 20 ng/dL	956	4.8%	492	4.9%	464	4.7%	1
Low-Density Lipoprotein, mg/dL	85.8	34.5	85.8	36.2	85.8	32.9	0
Triglycerides, mg/dL	145.5	111.2	145.5	109.7	145.6	112.5	0
High-Density Lipoprotein, mg/dL	43.2	14.2	43.3	14.5	43.2	13.9	1
Cholesterol, mg/dL	155.1	43.2	155.1	45.4	155.1	41.0	0
Sodium, mmol/L	138.9	3.3	138.9	3.4	138.9	3.1	0
Potassium, mmol/L	4.3	0.5	4.3	0.5	4.3	0.5	0
Creatinine, mg/dL	1.3	0.6	1.3	0.6	1.3	0.6	0
Calcium, mg/dL	9.2	0.5	9.2	0.6	9.2	0.5	0
Hemoglobin A1C, %	6.8	1.5	6.8	1.6	6.8	1.5	0
Ejection Fraction							
≤40%	5307	26.6%	2683	26.6%	2624	26.7%	0
>40%	7269	36.5%	3708	36.7%	3561	36.2%	1
Unknown	7349	36.9%	3704	36.7%	3644	37.1%	1

Abbreviations: SD: standard deviation; ASD: absolute standardized difference; IPTW: inverse probability of treatment weighting.

**Table 4:** All-cause death or hospitalization over 5 years of follow-up in the IPT-weighted cohort.

	# Events	# Patients at Risk	Event/Patients at Risk (%)	Mean (STD) Follow-Up Years	Median (IQR) Follow-Up Years	Total Person Years	Incidence Rate (per 1000 Person-Years)
Magnesium non-users	3013	9829	30.7%	0.85 (0.96)	0.70 (0.10–1.00)	8365.5	360.2
Magnesium users	2452	10096	24.3%	0.82 (0.95)	0.60 (0.10–1.00)	8234.1	297.8

Abbreviations: #, number of; IPT, inverse probability of treatment; STD, standard deviation; IQR: interquartile range.

## Data Availability

The datasets presented in this article are not readily available because of the sensitive nature of the data involved and the access to the dataset is restricted. Requests to access the datasets should be directed to the Washington DC VA Medical Center. Only qualified researchers trained in human subject confidentiality may request access by contacting via email at helen.sheriff@va.gov.

## Supplementary Materials

The following supporting information can be downloaded at: https://www.mdpi.com/article/10.3390/nu17233687/s1, Figure S1: Plots of ASDs before and after IPT-weighting.
