# Supplementary material for: Association Between Over-the-Counter Magnesium Supplement Use and Health Outcomes in Veterans with Newly Diagnosed Heart Failure
Source: Nutrients. 2025 Nov 25;17(23):3687. doi: 10.3390/nu17233687 (PMC12694171; doi:10.3390/nu17233687)
Supplement: Supplementary file 1 [file nutrients-17-03687-s001.zip › nutrients-3944541-supplementary.pdf]

Supplementary Figure S1: Plots of ASDs before and after IPT-weighting

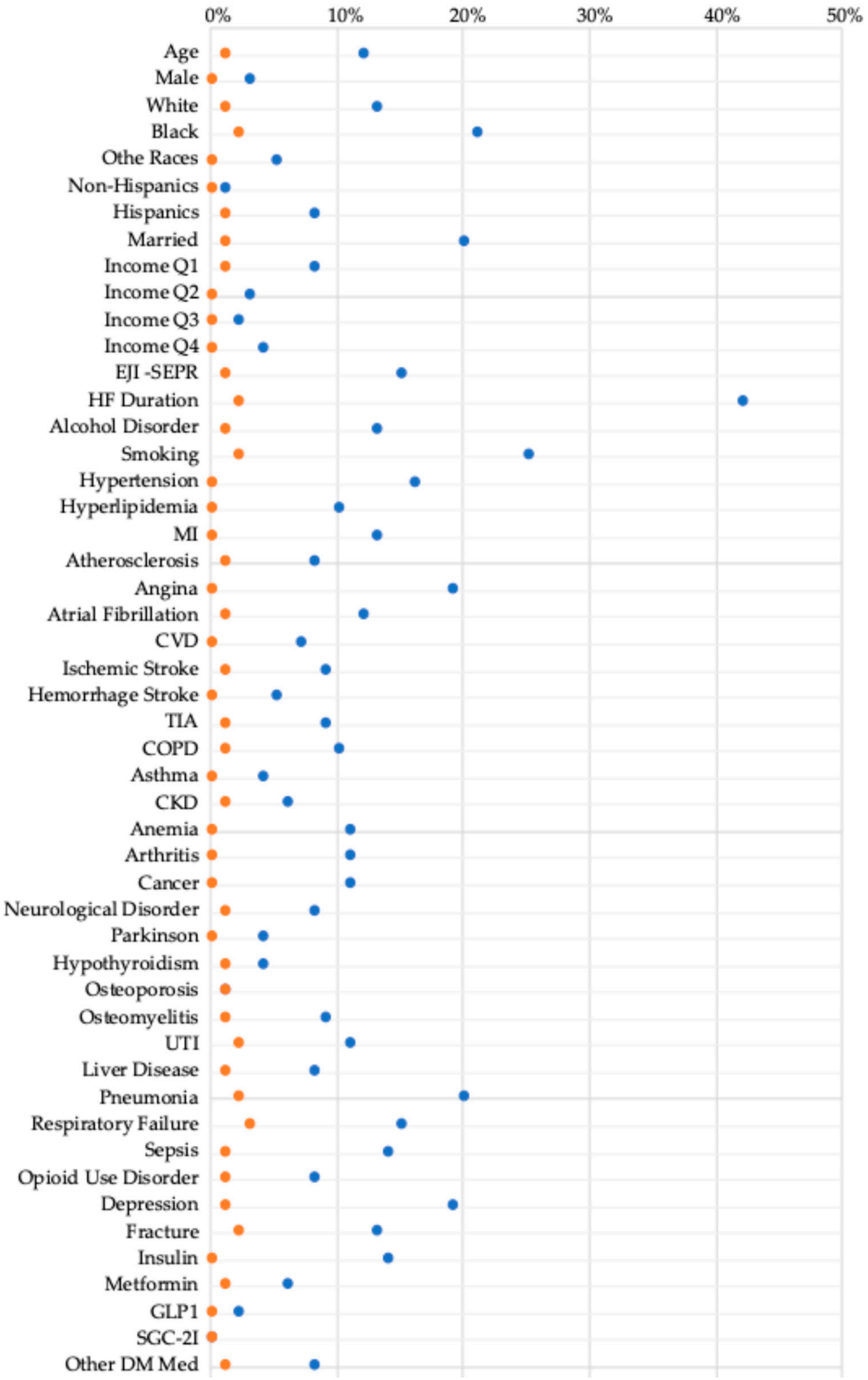

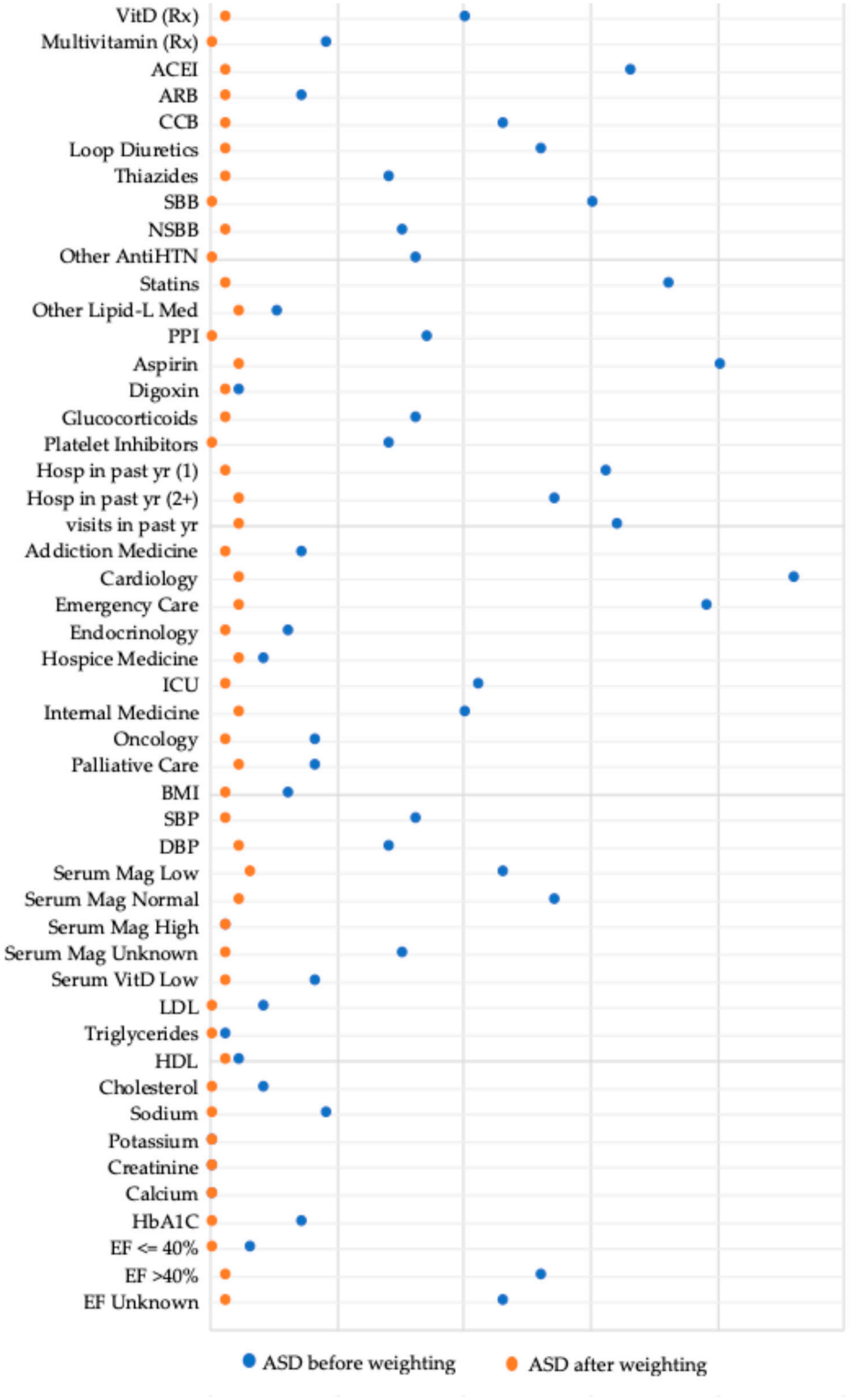

ASD: Absolute Standardized Difference; IPT: Inverse Probability Of Treatment; Q: Quartile; EJI-SEPR: Environmental Justice Index Social-Environmental Percentile Ranking; HF: Heart Failure; MI: Myocardial Infarction; CVD: Cardiac Valve Disease; TIA: Transient Ischemic Attack; COPD: Chronic Obstructive Pulmonary Disease; CKD: Chronic

Kidney Disease: UTI: Urinary Tract Infection; GLP1: Glucagon-Like Peptide-1; SGC-2I: Sodium-Glucose Cotrasporter-2 Inhibitors; Med: Medication; VitD: Vitamin D; ACEI: Angiotensin Converting Enzyme Inhibitor; ARB: Angiotensin Receptor Blocker; CCB: Calcium Channel Blocker; SBB: Selective Beta Blocker: NSBB: Non-Selective Beta Blocker: AntiHTN: Antihypertensive; Lipid-L: Lipid Lowering; PPI: Proton-Pump Inhibitors; Hosp: Hospitalization; yr: years; ICU: Intensive Care Unit; BMI: Body Mass Index; SBP: Systolic Blood Pressure; DBP: Diastolic Blood Pressure; Mag: Magnesium; LDL: Low-Density Lipoprotein; HDL: High-Density Lipoprotein; HbA1C: Hemoglobin A1C; EF: Ejection Fraction.
